# The Effects of Adjuvants on *Steinernema Carpocapsae* Efficacy Against *Chrysodeixis Includens* and Suspension Stability

**DOI:** 10.2478/jofnem-2025-0063

**Published:** 2026-01-31

**Authors:** Minling Zhang, Nathan R. Spaulding, Gadi V. P. Reddy, David I. Shapiro-Ilan

**Affiliations:** Southern Insect Management Research Unit, United States Department of Agriculture, Agricultural Research Service, Stoneville, MS 38776; Southeastern Fruit and Tree Nut Research Station, United States Department of Agriculture, Agricultural Research Service, Byron, GA 31008

**Keywords:** adjuvant, biological control, *Chrysodeixis includens*, entomopathogenic nematode, sedimentation, *Steinernema carpocapsae*

## Abstract

*Chrysodeixis includens* is a major soybean pest in the southern United States and has developed resistance to many insecticides. Entomopathogenic nematodes (EPNs) are potential alternative control tools. Above ground EPN applications are limited by environmental stresses; thus, we evaluated adjuvants to improve the performance of *Steinernema carpocapsae*. Laboratory assays showed that Xanthan & Tween enhanced EPN efficacy at 24 hr post application across all four exposure durations, whereas Barricade did not improve efficacy at the 1 hr exposure compared with EPNs without adjuvants. At 72 hr post application, adjuvant treatments achieved >90% mortality beginning at the 4 hr exposure duration, whereas EPNs without adjuvants reached >90% only at 24 hr. Greenhouse trials indicated efficacy improvements with Xanthan & Tween under some conditions, whereas Barricade did not. Optimization tests demonstrated that adding Tween to Barricade significantly improved EPN performance. Both Xanthan & Tween and Barricade & Tween reduced nematode sedimentation. Overall, adjuvant enhanced formulations increased EPN efficacy and stability, supporting their potential as a biological management tool for *C. includens*.

In the southern United States, *C. includens* (Lepidoptera: Noctuidae), commonly known as the soybean looper, is a prominent defoliator of soybean crops, with infestations typically peaking during the late growing season from July to September (Jost and Pitre, 2002; Allen et al., 2021). Resistance in *C. includens* to widely used chemical insecticides (Felland et al., 1990; Thomas and Boethel, 1994; Mascarenhas and Boethel, 1997) has been observed. This resistance, coupled with the adverse effects of chemical agents on natural enemy populations and their considerable environmental impact, necessitates the exploration of alternative pest control strategies.

Entomopathogenic nematodes (EPNs) have emerged as biological control agents capable of targeting various economically important insect pests (Shapiro-Ilan et al., 2017), including *C. includens* (Zhang et al., 2024, 2025). However, the efficacy of EPNs in aboveground applications is constrained by their susceptibility to desiccation and ultraviolet (UV) radiation (Gaugler and Boush, 1978; Glazer, 1992). Additionally, EPNs tend to settle at the bottom of storage containers, which can result in uneven distribution when using sprayers that lack automatic mixing or aeration.

Efforts to enhance EPN application on foliage in aboveground pest control have involved the use of adjuvants, including surfactants, thickeners, and their combinations (Schroer and Ehlers, 2005; Schroer et al., 2005a; Shapiro-Ilan et al., 2010, 2015, 2016; Hussein et al., 2012; Noosidum et al., 2016; Glover et al., 2025). Schroer et al. (2005a) evaluated various adjuvants and the combinations of surfactants and polymers to enhance the efficacy of *S. carpocapsae* (Weiser) (Nematoda: Rhabditida: Steinernematidae) against *Plutella xylostella* larvae L. (Lepidoptera: Plutellidae) and demonstrated that a combination of 0.3% xanthan gum or 0.3% potassium alginate and 0.3% specific surfactants significantly enhanced *S. carpocapsae* efficacy and reduced infection time in the laboratory assays. A fire-retardant gel Barricade has also been reported to improve the effectiveness of *S. carpocapsae* in controlling the lesser peachtree borer, *Synanthedon pictipes* (Grote & Robinson) (Lepidoptera: Sesiidae), for aboveground applications in peach orchards, either through separate or combined applications (Shapiro-Ilan et al., 2010, 2016). Noosidum et al. (2016) later found that when *S. carpocapsae* and *Steinernema* sp. Isolate K8 (Thai isolate) was sprayed onto kale leaf discs, EPN survival rates 3 hr after incubation were significantly higher in the presence of 0.25% and 0.5% Barricade than EPNs in tap water alone. They also found that Barricade enhanced the virulence of *S. carpocapsae* against *Spodoptera litura* (F.) (Lepidoptera: Noctuidae) larvae. Wu et al. (2023) evaluated the effects of adjuvants, including Barricade (1.0% and 2.0%), on the protection of *S. carpocapsae* infective juveniles (IJs) from UV irradiation by exposing IJs to UV (254 nm) for 0 min, 10 min, and 20 min. Barricade, especially 2% Barricade, significantly improved IJ viability after UV treatment. Elgar et al. (2025) further confirmed the UV-protective properties of 2% Barricade and demonstrated that xanthan gum also provides UV protection.

In addition to enhancing the efficacy of EPNs, adjuvants have also been used to reduce EPN sedimentation in aqueous suspensions (Peters and Backes, 2003; Schroer et al., 2005b; Van Niekerk and Malan, 2015). Peters and Backes (2003) reported that 0.4% carboxymethylcellulose reduced the settling speed of *S. feltiae* (nemaplus®) from 0.58 cm/min to 0.02 cm/min. Schroer et al. (2005b) demonstrated that 0.05% xanthan gum, 0.1% potassium alginate, and 0.2%–0.3% guar gum significantly reduced the sedimentation of *S. carpocapsae* compared to water. Later, Van Niekerk and Malan (2015) evaluated the polymer product Zebaw and xanthan gum at various concentrations for their ability to reduce sedimentation of *Heterorhabditis zealandica* Poinar (Nematoda: Rhabditida: Heterorhabditidae) in water. Their findings indicated that xanthan gum at 0.1% and 0.2% effectively reduced sedimentation for up to 60 min. In contrast, Zebaw at 0.3% significantly delayed sedimentation for up to 30 min.

Our previous studies identified three adjuvant treatments – dish soap, a mixture of dish soap and vegetable oil, and Southern Ag Surfactant – as effective in enhancing the efficacy of *S. carpocapsae* against fifth instar larvae of *C. includens* under certain conditions (Zhang et al., 2025). These adjuvants are suitable for use when settling is not a concern, such as when small volumes of EPN suspensions are used in smaller containers or hand sprayers. This is because the spraying process is typically completed before significant EPN settling occurs, and shaking during application is manageable. However, when large volumes of EPN suspensions are used, the application may take longer to complete. If the sprayers lack automatic mixing or aeration, these adjuvants may underperform because of limited suspension stability. The objective here is to identify adjuvants that (1) improve biocontrol efficacy, and (2) provide stability in suspension. Xanthan & Tween were included based on previous laboratory findings indicating their potential in both categories mentioned above, although their use as an aboveground protectant in field conditions remains limited. Since prior studies (Schroer et al., 2005b; Van Niekerk and Malan, 2015) have evaluated only xanthan gum for suspension stability, we incorporated xanthan and Tween in this study to assess their performance in this context. Barricade was selected due to its extensive use as an aboveground protectant and its demonstrated potential to shield IJs from environmental stressors. We hypothesize that Barricade may also improve suspension stability, thereby enhancing the efficacy of EPNs for managing aboveground insect pests, such as *C. includens*.

## Materials and Methods

### Insect colony

Eggs of *C. includens* were procured from Benzon Research (Carlisle, PA). Upon hatching, larvae were reared under controlled conditions at 27°C with a 14:10 light-dark photoperiod and 70–80% relative humidity (RH) in environmental chambers (Percival Scientific, Perry, IA). Larvae were reared on an ARS soybean and wheat germ-based diet developed for *Heliothis virescens* (F.) (Lepidoptera: Noctuidae), following the protocol described by Blanco et al. (2009a, 2009b).

### EPN culture

The *S. carpocapsae* (all strain) used in this study was obtained from the USDA-ARS EPN Laboratory in Byron, Georgia, and cultured in the last instars of *Galleria mellonella* L. (Lepidoptera: Pyralidae) larvae sourced from Josh's Frogs (Owosso, MI) (Zhang et al., 2024). IJs were extracted using White traps (Kaya and Stock, 1997) and stored at 13°C for no >2 weeks before use for the experiments. The IJs were then counted, and their concentrations were adjusted by diluting the IJs with tap water 1 day prior to each experiment.

### Adjuvants

Three adjuvants were employed in this investigation: xanthan gum, a thickening agent derived from *Xanthomonas campestris* (Sigma-Aldrich Inc., St. Louis, MI); Tween® 80, a surfactant comprising polyoxyethylenesorbitan monooleate (Sigma-Aldrich Inc.); and Barricade, a thickening fire retardant gel composed of sodium polyacrylate and modified vegetable oil (Barricade® II Fire Gel, International, Inc., Hobe Sound, FL). Adjuvants were prepared 1 day before application and mixed with EPN suspensions immediately before use.

### Effect of *S. carpocapsae* with adjuvants on fifth instars of *C. includens* under laboratory conditions

To assess the influence of adjuvants on EPN *S. carpocapsae* efficacy against fifth instars of *C. includens*, three EPN treatments were evaluated across four exposure durations. The EPN treatments consisted of: (1) 0.3% (equivalent to 3 g/L) xanthan gum combined with 0.3% Tween® 80 (Schroer et al., 2005a, 2005b; hereafter referred to as Xanthan & Tween), (2) 0.3% Barricade (Noosidum et al., 2016), and (3) no adjuvants. Additionally, controls without EPNs were run for each treatment for each exposure duration. Each treatment was replicated three times with 10 insects per replicate, and the entire experiment was conducted twice. Experimental procedures followed the methodology outlined by Zhang et al. (2025). Larvae were placed individually into 60 × 15 mm petri dishes lined with a layer of filter paper (#2, Advantec MFS, Kashiwa, Chiba, Japan) and treated with 1 mL of EPN suspension at 100 IJ/mL or with a solution without EPNs. The dishes were then placed in a dark incubator set at 25°C and with a RH of 85% or higher for the duration of the experiment. Exposure durations of 1 hr, 4 hr, 8 hr, and 24 hr were tested. After each respective exposure period, insects were transferred from the petri dishes to diet cups and returned to the same incubator. Larval mortality was recorded daily for 72 hr post-application.

### Effect of *S. carpocapsae* with adjuvants on fifth instars of *C. includens* under greenhouse conditions

The EPN treatments described above were also evaluated in a greenhouse with limited control of temperature and humidity. Since no insect toxicity was observed with the adjuvants, only water was used as the control in the greenhouse experiment. The EPN concentration for all treatments was standardized at 10 IJs/cm^2^ (equivalent to 450 IJs/mL in a 30 mL volume). Each treatment was replicated three times with 10 insects per replicate, and tested across four independent trials. Soybean plants were cultivated in 1.9-liter pots, with two plants per pot. Each replicate consisted of 8–10 plants. The plants used were 8–12 weeks old and had an average foliar area of approximately 1,350 cm^2^. Experimental procedures followed the method described by Zhang et al. (2025), except that no additional water was applied post-application. Trials were run in the early morning. The temperature and RH at the beginning of treatment, as well as the respective daily minimums and maximums, were recorded using a digital hygrometer and thermometer. Ten laboratory-reared fifth instars of *C. includens* were placed onto the plants, followed by the application of 30 mL of either EPN suspension or control water using a 60 mL pump sprayer in such a way that the front and back of leaves were treated. Each replicate's plant grouping was subsequently placed individually in an insect cage (0.4 m × 0.4 m × 0.6 m with mesh holes <0.8 mm) (Insect and Butterfly Habitat Cage Terrarium, Restcloud brand, China) to prevent insect escape. After 24 hr, larvae were removed from the plants and placed individually in diet cups, which were then incubated at room temperature (~22°C). Insect mortality was recorded daily until 72 hr post-application.

### Optimization of the effect of *S. carpocapsae* with Barricade on fifth instars of *C. includens* under greenhouse conditions

This experiment was conducted in response to the lack of observed efficacy of Barricade in previous greenhouse trials. The experiment included five EPN treatments: (1) 1.0% Barricade combined with 0.3% Tween® 80 (hereafter referred to as 1.0% Barricade & Tween), (2) 0.3% Barricade combined with 0.3% Tween® 80 (hereafter referred to as 0.3% Barricade & Tween), (3) 1.0% Barricade, (4) 0.3% Barricade, and (5) no adjuvants. A water control was also included. Each treatment was replicated three times, and the entire experiment was conducted across three independent trials. The EPN concentration was 10 IJs/cm^2^. The application methods mirrored those used in previous experiments.

### Effect of adjuvants on *S. carpocapsae* sedimentation under laboratory conditions

Based on the greenhouse results, Xanthan & Tween and Barricade & Tween treatments were selected for further evaluation of their effects on IJ sedimentation. Sedimentation was measured using a method adapted from Schroer et al. (2005b), initially described by Peters and Backes (2003). Xanthan gum was tested at concentrations of 0.1%, 0.2%, and 0.3%, while Barricade was tested at 0.3%, 1.0%, and 2.0%; each concentration was combined with 0.3% Tween (hereafter referred to as 0.1%, 0.2% or 0.3% Xanthan & Tween, 0.3%, 1.0%, or 2.0% Barricade & Tween). The control treatment consisted of EPNs without any adjuvants. Each treatment included three replicates, with each replicate containing a 500 mL EPN suspension (1000 IJs/mL) held in a plastic container (75.7 mm height × 98.4 mm diameter). Sedimentation was assessed by collecting three 100 μL samples from each replicate at a depth of 2 cm at 0 min, 5 min, 10 min, 15 min, 30 min, and 60 min following cessation of stirring. The mean value of the three 100 μL samples was used for each replicate to reduce the variability between replicates. For each observation time, the percentage of IJs per replicate was calculated by dividing the observed IJ count by the mean IJ count obtained at time zero (immediately following stirring). The entire experiment was repeated on a separate date using a different batch of IJs.

### Data analysis

Percentages for insect mortality and IJ percentages were normalized using an arcsine square root transformation before analysis. The effects of *S. carpocapsae* with adjuvants on *C. includens* under laboratory conditions were evaluated using a two-way ANOVA, with treatment and exposure duration as main factors (Noosidum et al., 2016). Insect mortality at 24 hr and 72 hr post-application was analyzed separately. In the greenhouse experiments, a one-way ANOVA was used to assess the effect of adjuvants on insect mortality. IJ percentages in the sedimentation test were evaluated using a two-way ANOVA, with observation time and adjuvant concentration as the main factors. Data for Xanthan & Tween, as well as Barricade & Tween, were analyzed separately. Tukey's HSD test in JMP version 15.0.0 was used to compare treatment means, and the differences were considered significant when *P* ≤ 0.05. All tables are presented with untransformed treatment means.

## Results

### Effect of *S. carpocapsae* with adjuvants on fifth instars of *C. includens* under laboratory conditions

As the results at 48 hr closely resembled those at 72 hr, only the insect mortality for 24 hr and 72 hr is presented in Table 1. At 24 hr post-application, insect mortality was significantly influenced by treatment (six levels: three EPN treatments with Xanthan & Tween, Barricade, or no adjuvants, and three controls without EPNs; *F* = 105.46, df = 5, 120, *P* < 0.0001) and EPN exposure duration (four levels: 1 hr, 4 hr, 8 hr, and 24 hr; *F* = 6.89, df = 3, 120, *P* = 0.0003), with a significant interaction between the two factors (*F* = 2.29, df = 15, 120, *P* = 0.0072). Similarly, at 72 hr post-application, insect mortality was significantly affected by treatment (*F* = 671.70, df = 5, 120, *P* < 0.0001) and exposure duration (*F* = 16.65, df = 3, 120, *P* < 0.0001), with a significant interaction between the two factors (*F* = 5.28, df = 15,120, *P* < 0.0001). Because of these significant interactions, simple effects analyses were conducted to examine how each factor influenced insect mortality across the levels of the other.

**Table 1:** Mortality (%±SEM) of fifth instars of *C. includens* following 1 hr, 4 hr, 8 hr, or 24 hr of exposure to *S. carpocapsae* (all strain) with adjuvants, assessed at 24 hr and 72 hr post-application under laboratory conditions (25°C, 85% RH).

**Exposure (hr)**	**Treatment**	**24 hr mortality**	**72 hr mortality**
1	Sc + X + T^a^	48.3 ± 9.8 A, a	88.3 ± 3.1 A, b
	Sc + B	26.7 ± 6.7 AB, b	73.3 ± 8.8 AB, b
	Sc	8.3 ± 1.7 BC, a	53.3 ± 6.1 B, b
	Water + X + T	0.0 ± 0.0 C, a	1.7 ± 1.7 C, a
	Water + B	0.0 ± 0.0 C, a	0.0 ± 0.0 C, a
	Water	0.0 ± 0.0 C, a	0.0 ± 0.0 C, a

4	Sc + X + T	53.3 ± 11.7 A, a	100.0 ± 0.0 A, a
	Sc + B	56.7 ± 3.3 A, a	93.3 ± 2.1 A, ab
	Sc	5.0 ± 2.2 B, a	68.3 ± 8.7 B, b
	Water + X + T	0.0 ± 0.0 B, a	0.0 ± 0.0 C, a
	Water + B	0.0 ± 0.0 B, a	0.0 ± 0.0 C, a
	Water	0.0 ± 0.0 B, a	1.7 ± 1.7 C, a

8	Sc + X + T	56.7 ± 14.3 A, a	100.0 ± 0.0 A, a
	Sc + B	56.7 ± 5.6 A. a	96.7 ± 2.1 A, ab
	Sc	3.3 ± 2.1 B, a	76.7 ± 7.1 B, b
	Water + X + T	0.0 ± 0.0 B, a	1.7 ± 1.7 C, a
	Water + B	0.0 ± 0.0 B, a	1.7 ± 1.7 C, a
	Water	0.0 ± 0.0 B, a	0.0 ± 0.0 C, a

24	Sc + X + T	80.0 ± 11.3 A, a	100.0 a ± 0.0 A, a
	Sc + B	71.7 ± 8.3 A, a	98.3 a ± 1.7 A, a
	Sc	26.7 ± 9.9 B, a	96.7 a ± 2.1 A, a
	Water + X + T	0.0 ± 0.0 C, a	0.0 ± 0.0 B, a
	Water + B	0.0 ± 0.0 C, a	0.0 ± 0.0 B, a
	Water	0.0 ± 0.0 C, a	0.0 ± 0.0 B, a

The same uppercase or lowercase letters indicate no significant differences among treatments within the same exposure duration or across exposure duration at the same treatment, respectively at *P* > 0.05 (Tukey's HSD test).

a Sc = *Steinernema carpocapsae* (all strain), X = 0.3% xanthan gum, T = 0.3% Tween® 80, B = 0.3% Barricade®.

RH, relative humidity.

Treatment effects on insect mortality within each exposure duration were indicated by uppercase connecting letters in Table 1. Following 1 hr of EPN exposure, the Xanthan & Tween treatment significantly increased insect mortality at both 24 hr and 72 hr post-application compared to EPNs without adjuvants, whereas the 0.3% Barricade treatment had no significant effect. For exposure durations of 4 hr and 8 hr, both the Xanthan & Tween and Barricade treatments significantly enhanced insect mortality compared to EPNs without adjuvants at both 24 hr and 72 hr post-application. When exposure duration increased to 24 hr, however, both treatments significantly increased insect mortality compared to EPNs without adjuvants at 24 hr post-application, but neither demonstrated a significant effect at 72 hr post-application. Additionally, only EPNs with adjuvant treatments resulted in significantly higher insect mortality than no-EPN controls at 24 hr post-application across exposure durations of 1 hr, 4 hr, and 8 hr. In contrast, all EPN treatments – both with and without adjuvants – caused significantly higher insect mortality than no-EPN controls at 24 hr post-application after 24 hr of exposure, and at 72 hr post-application across all exposure durations (1 hr, 4 hr, 8 hr, and 24 hr).

The effects of EPN exposure duration on insect mortality within each treatment were denoted by lowercase connecting letters in Table 1. At 24 hr post-application, insect mortality for 1 hr, 4 hr, 8 hr, and 24 hr exposure durations ranged from 48.3%–80.0% with Xanthan & Tween, 26.7%–71.7% with Barricade, and only 8.3%–26.7% with EPNs applied without adjuvants. Insect mortality did not differ significantly among exposure durations in the Xanthan & Tween or EPNs without adjuvant treatments, whereas in the Barricade treatment, mortality at 4 hr, 8 hr, and 24 hr of exposure was significantly higher than at 1 hr. At 72 hr post-application, insect mortality ranged from 88.3%–100.0% with Xanthan & Tween, 73.3 hr–98.3% with Barricade, and 53.3 hr–96.7% with EPNs without adjuvants. Both Xanthan & Tween and Barricade treatments produced >90% mortality at 4 hr, 8 hr, and 24 hr of exposure. For Xanthan & Tween, mortality at 4 hr, 8 hr, and 24 hr of exposure was significantly higher than at 1 hr; for Barricade, significant differences were observed only at 24 hr. In contrast, EPNs without adjuvants reached >90% mortality only at 24 hr of exposure, which was significantly higher than at 1 hr, 4 hr, and 8 hr.

### Effect of *S. carpocapsae* with adjuvants on fifth instars of *C. includens* under greenhouse conditions

Since no insect mortality was observed at 24 hr across all four trials, and the results at 48 hr closely mirrored those at 72 hr, only the 72 hr post-application mortality is presented in Table 2. No significant differences in insect mortality were observed among EPN treatments with or without adjuvants in trial 1 (17–30°C, RH 79%–99%) and trial 3 (15–28°C, RH 67%–99%). In contrast, in trials 2 (13–27°C, RH 55%–84%) and 4 (12–29°C, RH 48%–85%), the Xanthan & Tween treatment significantly increased insect mortality relative to the EPNs without adjuvant control in both trials, whereas the Barricade treatment showed no significant effect. Across all trials, EPN treatments consistently resulted in higher mortality compared to the water controls.

**Table 2:** Mortality (%±SEM) of fifth instars of *C. includens* caused by *S. carpocapsae* (all strain) with adjuvants 72 hr post-application under greenhouse conditions.

Trial	1	2	3	4
Temperature (°C)^a^	20 (17–30)	18 (13–27)	19 (15–28)	15 (12–29)
RH (%)^a^	84 (79–99)	66 (55–84)	71 (67–99)	71 (48–85)
Plant stage	Beginning pod	Poding	Poding	Poding
High (cm)	31.2	38.1	38.1	39.4

Sc + X + T^b^	100.0 ± 0.0	86.7 ± 3.3 a	96.7 ± 3.3 a	100.0 ± 0.0 a
Sc + B	100.0 ± 0.0	46.7 ± 6.7 b	96.7 ± 3.3 a	73.3 ± 8.8 b
Sc	100.0 ± 0.0	40.0 ± 5.8 b	96.7 ± 3.3 a	56.7 ± 12.0 b
Water	0.0 ± 0.0	0.0 ± 0.0 c	0.0 ± 0.0 b	0.0 ± 0.0 c

*F*-value (3, 8)		19.71	62.07	61.16
*P*-value		0.0005	<0.0001	<0.0001

Mean values within a column followed by the same letter are not significantly different at *P* > 0.05 (Tukey's HSD test).

a Numbers outside parentheses were the temperature or RH (relative humidity) at the beginning of application, and numbers inside parentheses were the lowest-highest temperature or RH during the first 24 hr of the trial.

b Sc = *Steinernema carpocapsae* (all strain), X = 0.3% xanthan gum, T = 0.3% Tween® 80, B = 0.3% Barricade®.

### Optimization of the effect of *S. carpocapsae* with Barricade on fifth instars of *C. includens* under greenhouse conditions

No insect mortality was observed at 24 hr in trial 1. In trials 2 and 3, treatments with 0.3% or 1.0% Barricade & Tween resulted in insect mortality of no >6.7% at 24 hr, which were not significantly different from the water control. Since insect mortality at 48 hr closely mirrored that at 72 hr, only 72-hr mortality is presented in Table 3. In trial 1 (13–22°C, 53%–88% RH), all EPN treatments significantly increased insect mortality compared to the water control. The insect mortality in the 1.0% Barricade & Tween treatment was significantly higher than in the 1.0% Barricade without Tween treatment. Similarly, the 0.3% Barricade & Tween treatment resulted in significantly higher insect mortality than the 0.3% Barricade treatment. Notably, only the 1.0% Barricade & Tween treatment significantly enhanced insect mortality compared to the EPN without Tween treatment. In trial 2 (16–36°C, 16%–78% RH), all EPN treatments, except for the 0.3% Barricade treatment, significantly increased insect mortality compared to the water control. Both 1.0% and 0.3% Barricade & Tween treatments significantly enhanced insect mortality compared to the corresponding Barricade treatments without Tween. However, neither showed a significant difference when compared to the EPN without adjuvant treatment. In trial 3 (19–40°C, 25%–84% RH), only the 1.0% and 0.3% Barricade & Tween treatments showed significantly higher insect mortality than the water control; no significant differences were observed among the EPN treatments.

**Table 3:** The effect of Barricade optimization on mortality (%±SEM) of *C. includens* fifth instars caused by *S. carpocapsae* (All strain) 72 hr post-application under greenhouse conditions.

Trial	1	2	3
Temperature (°C)^a^	20 (13–22)	18 (16–36)	20 (19–40)
RH (%)^a^	54 (53–88)	47 (16–78)	53 (25–84)
Plant stage	Fifth trifoliolate	Sixth trifoliolate	Seventh trifoliolate
High (cm)	33	36	46

Sc + 1.0%B + T^b^	90.0 ± 5.8 a	40.0 ± 5.8 a	26.7 ± 3.3 a
Sc + 0.3%B + T	83.3 ± 6.7 ab	36.7 ± 6.7 a	44.1 ± 18.1 a
Sc + 1.0%B	56.7 ± 3.3 bc	10.0 ± 0.0 b	10.8 ± 0.8 ab
Sc + 0.3%B	36.7 ± 6.7 c	6.7 ± 3.3 bc	13.7 ± 8.8 ab
Sc	63.3 ± 3.3 bc	17.8 ± 3.4 ab	20.0 ± 0.0 ab
Water	0.0 ± 0.0 d	0.0 ± 0.0 c	0.0 ± 0.0 b

*F*-value (5, 12)	38.60	18.73	5.43
*P*-value	<0.0001	<0.0001	0.0077

Mean values within a column followed by the same letter are not significantly different at *P* > 0.05 (Tukey's HSD test).

a Numbers outside parentheses were the temperature or RH (relative humidity) at the beginning of application, and numbers inside parentheses were the lowest-highest temperature or RH during the first 24 hr of the trial.

b Sc = *Steinernema carpocapsae* (all strain), B = Barricade®, T = 0.3% Tween® 80.

IJs, infective juveniles.

### Effect of adjuvants on *S. carpocapsae* sedimentation under laboratory conditions

**Figure 1:**
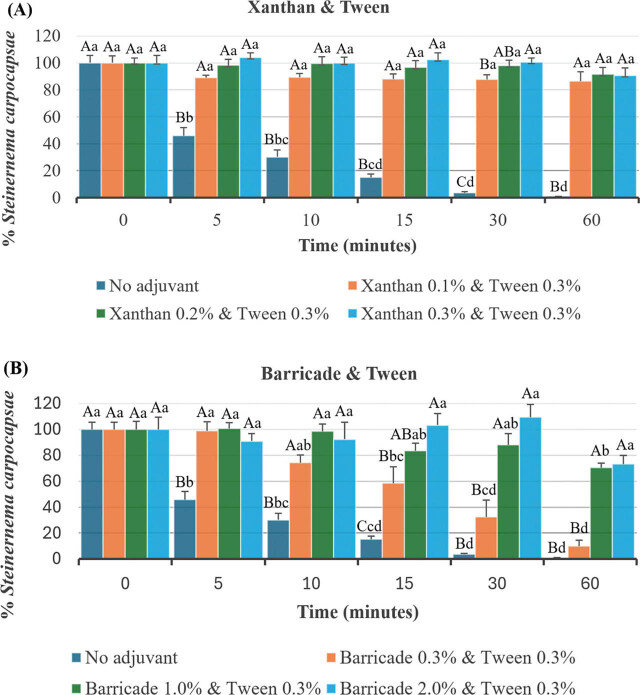
Mean (±SEM) percentage IJs of S. carpocapsae observed at a 2 cm depth following cessation of stirring at set time intervals for treatments of Xanthan & Tween (A) and Barricade & Tween (B) at different concentrations. The same uppercase or lowercase letters indicate no significant differences among concentrations within the same time interval or across time intervals at the same concentration, respectively (Tukey's HSD test, α > 0.05). IJs, infective juveniles.

In the Xanthan & Tween treatments, both observation time (six levels: 0 min, 5 min, 10 min, 15 min, 30 min and 60 min; *F* = 26.62; df = 5, 120; *P* < 0.0001) and adjuvant concentration (four levels: no adjuvant, and 0.1%, 0.2%, and 0.3% xanthan gum each combined with 0.3% Tween; *F* = 307.35; df = 3, 120; *P* < 0.0001) had significant effects on IJ sedimentation. A significant interaction between observation time and adjuvant concentration was also observed (*F* = 15.35; df = 15, 120; *P* < 0.0001). Due to this significant interaction, a simple effects analysis was conducted to examine how each factor influenced sedimentation across the levels of the other (Fig. 1A). Across the 5- to 60-min intervals, all Xanthan & Tween treatments significantly slowed IJ sedimentation compared to the no-adjuvant control. Among these treatments, the 0.3% Xanthan & Tween treatment resulted in significantly reduced IJ sedimentation compared to the 0.1% Xanthan & Tween treatment after 30 min. Over the 60 min observation period, IJs in all three Xanthan & Tween treatments showed no significant changes from their initial percentages. In contrast, the no-adjuvant control displayed rapid sedimentation, with only 45.2% of the initial IJ count remaining after 5 min and just 0.9% after 60 min.

In the Barricade & Tween treatments, both observation time (six levels: 0 min, 5 min, 10 min, 15 min, 30 min and 60 min; *F* = 33.55; df = 5, 120; *P* < 0.0001) and adjuvant concentration (four levels: no adjuvant, and 0.3%, 1.0%, and 2.0% Barricade each combined with 0.3% Tween; *F* = 92.13; df = 3, 120; *P* < 0.0001) had significant effects on IJ sedimentation. A significant interaction between observation time and adjuvant concentration was also detected (*F* = 7.30; df = 15, 120; *P* < 0.0001). To investigate these interactions in greater detail, a simple effects analysis was conducted to determine how each factor impacted sedimentation across the varying levels of the other factor. (Fig. 1B). All Barricade & Tween treatments significantly reduced IJ sedimentation compared to the no adjuvant control at the 5–15 min intervals. However, only the 1.0% and 2.0% Barricade & Tween treatments maintained significant reductions at both 30 min and 60 min. In the 0.3% Barricade & Tween treatment, IJ percentages remained stable up to the 10-min observation period but declined significantly from the 15- to 60-min intervals relative to their initial levels. By contrast, IJ percentages in the 1.0% and 2.0% Barricade & Tween treatments remained stable throughout the 30 min observation period. At 60 min, however, only the 2.0% treatment maintained IJ percentages without a significant decline from its initial IJ percentage.

## Discussion

This study highlights that both adjuvant treatments, Xanthan & Tween and Barricade, can reduce the time required for *S. carpocapsae* to act and improve its efficacy against fifth instars of *C. includens* under laboratory conditions. Under greenhouse conditions, Xanthan & Tween significantly enhanced EPN efficacy, as did the addition of Tween to the Barricade treatment. Additionally, laboratory results on *S. carpocapsae* sedimentation indicate that adjuvant treatments, including Xanthan & Tween, and Barricade & Tween, can effectively reduce the sedimentation on *S. carpocapsae*.

Xanthan gum functions effectively as a thickener, improving adhesion and preventing rapid drying. Meanwhile, Tween acts as a versatile non-ionic surfactant and emulsifier. Schroer et al. (2005a) found that adding xanthan gum doubled insect mortality at 80% RH and increased it fivefold at 60% RH. Furthermore, mixtures of 0.3% xanthan gum with 0.3% surfactants, including Tween, further enhanced efficacy. The adjuvant treatments showed greater virulence, resulting in higher insect mortality than the no-adjuvant controls. Our current findings obtained under laboratory conditions show similar results, where insect mortality observed 24 hr post-application was significantly higher in the Xanthan & Tween treatments than in those without them across all exposure times (1 hr, 4 hr, 8 hr, and 24 hr). These findings indicate that Xanthan & Tween treatments reduced the time required for *S. carpocapsae* to lethally affect *C. includens* larvae, compared with application in water alone. Thus, at 72 hours post application, EPN with Xanthan & Tween caused >90.0% insect mortality at 4 hr, 8 hr, and 24 hr of exposure, whereas EPNs without adjuvant reached > 90.0% only at 24 hr. In our greenhouse experiment, while Xanthan & Tween treatments enhanced the effectiveness of *S. carpocapsae* against fifth instars of *C. includens*, xanthan gum was applied to foliage not as a fine mist but in the form of clumps. Despite evaluating various hand sprayers, none were able to uniformly disperse the suspension. For this small-scale experiment, the suspension was meticulously applied to the leaves to achieve maximum uniformity. However, future large-scale field applications will require the adoption of more advanced and efficient sprayer technologies.

Barricade is a fire-resistant product designed to protect homes and properties from wildfires. When mixed with water, it forms a thermal-protective coating. Shapiro-Ilan et al. (2010) evaluated the use of a full rate of Barricade (approximately 4%) as a secondary application following the treatment of *S. carpocapsae* to control the lesser peachtree borer, *S. pictipes*. This approach reduced *S. pictipes* populations compared to the controls and an EPN-only treatment, with the Barricade treatment having larval survival rates of 30% in 2008 and 0% in 2009. In 2013 and 2014, Shapiro-Ilan et al. (2016) tested 2.0% and 4.0% of Barricade combined with *S. carpocapsae* as a single spray to manage *S. pictipes*. When compared to the chemical standard, chlorpyrifos, suppression of *S. pictipes* in both Barricade plus EPN treatments was comparable. Notably, the 2% application was the most effective treatment, achieving significant control of *S. pictipes* compared with the untreated control in both years of the study. Noosidum et al. (2016) reported similar effectiveness on kale plants under greenhouse conditions. Barricade treatments of 0.25% and 0.5% mixed with *S. carpocapsae* resulted in a respective 66.0% and 61.5% mortality of larval *S. litura*, which were significantly higher than the 29.5% observed in the EPN treatment lacking Barricade. However, for *P. xylostella* larvae, mortality from the 0.25% and 0.5% Barricade treatments did not reach a significant level compared with *S. carpocapsae* plus tap water. In our experiments, 0.3% Barricade alone improved the efficacy of *S. carpocapsae* against insects under laboratory conditions; however, it did not enhance efficacy in greenhouse trials. The addition of Tween to the Barricade treatments resulted in enhanced efficacy of *S. carpocapsae.*

Since the no-EPN Xanthan & Tween control did not exhibit toxicity toward *C. includens* larvae in our laboratory experiment, Tween may have enhanced the effectiveness of the Barricade through its surfactant function. By lowering the surface tension of water, Tween improves coverage on plant surfaces and promotes closer contact between EPNs and insect cuticles, thereby facilitating EPN penetration into insect hosts. However, the adjuvant enhancement was limited by environmental conditions, with low humidity and high temperatures reducing its effectiveness.

Elgar et al. (2025) evaluated the virulence of *S. carpocapsae* suspended in five gel-based adjuvants against *G. mellonella* larvae, including 2.0% Barricade and 1.0% xanthan gum. EPNs were sprayed onto peach bark and exposed to natural sunlight for 1 hr, 2 hr, and 3 hr. Both 2.0% Barricade and 1.0% xanthan gum treatments resulted in significantly higher insect mortality compared to EPNs without adjuvants across all exposure durations. However, after 2 hr and 3 hr of exposure, 2.0% Barricade retained higher virulence than xanthan gum, although this difference did not reach statistical significance after 3 hr. In our greenhouse experiments, Xanthan & Tween performed better than 0.3% Barricade. Except for the inherent differences between the two materials, this may be attributed to the addition of Tween to xanthan gum, differences in adjuvant concentrations, and the greenhouse environment itself.

Although insect mortality was observed at 24 hr in laboratory experiments following just 1 hr of exposure to EPNs, only low levels of mortality (most were 0.0%, with a maximum of 6.7%) were recorded in the greenhouse trials. One possible explanation is the relatively low nighttime temperatures in greenhouse trials, with minimum temperatures ranging from 12°C to 19°C, which may have slowed the development of symbiotic bacteria within the insect hosts. Additionally, the presence of plants and the complex greenhouse conditions may have influenced the ability of EPNs to locate and infect the insects, potentially requiring more time for successful host penetration.

Schroer et al. (2005b) reported that 5 min after stirring had ceased, 50% of the IJs had passed beyond a depth of 2 cm, whereas *S. carpocapsae* in water only and 0.05% xanthan gum successfully prevented sedimentation for 60 min. Van Niekerk and Malan (2015) further confirmed that both 0.1% and 0.2% xanthan gum effectively reduced the sedimentation of *H. zealandica*. However, the percentages of IJs for both 0.1% and 0.2% xanthan gum at 60 min were significantly lower compared to their initial percentages. Our current findings further confirm that combining xanthan gum and Tween maintains its effectiveness in reducing the sedimentation of *S. carpocapsae*. Moreover, the IJ percentages at 60 min for both 0.1% and 0.2% Xanthan & Tween treatments did not differ significantly from the initial IJ percentages. While the addition of Tween might have contributed to differences in sedimentation observed between our experiments and those of Van Niekerk and Malan (2015), another impacting factor could be the size differential of the IJs in each study (685 μm for *H. zealandica* and 585 μm for *S. carpocapsae*, source: Stock and Hunt (2005)). As reported by Beck et al. (2013), larger IJs tend to settle more rapidly than smaller ones.

Our current study supports our previous experiments (Zhang et al., 2025), which demonstrated that the adjuvant-enhancing effect varies under different RH and temperature conditions. Therefore, to fully understand the effects of adjuvants, their performance under various conditions should be evaluated. At the same time, the outcome suggests that selecting optimal conditions to maximize biocontrol efficacy is crucial.

Xanthan gum has been less extensively used as an aboveground protectant under field conditions compared to Barricade. Recently, Metwally et al. (2025) evaluated two species of EPNs, *S. feltiae* and *H. bacteriophora* (HP88 strain), mixed with xanthan gum or Tween 80 to control onion thrips, *Thrips tabaci* Lindeman (Thysanoptera: Thripidae). Their results indicated that, under semi-field conditions, both xanthan gum and Tween 80 enhanced the persistence of *S. feltiae* on onion plants. Furthermore, in their field trials, combining *S. feltiae* or *H. bacteriophora* with 0.3% xanthan gum or Tween 80 individually reduced thrips populations on onion leaves by 28.02%–63.24% within 3 days. In addition, our previous studies demonstrated that surfactants can enhance the efficacy of EPNs against the first instars of *Helicoverpa zea* in corn plants under field conditions (Zhang et al., 2024), as well as against fifth instar larvae of *C. includens* under certain greenhouse conditions. Taken together, these findings highlight the need for further research to evaluate the effects of thickening-enhancing adjuvants combined with surfactants – specifically xanthan gum with Tween 80 and Barricade with Tween 80 – under field conditions.
